# Occurrence of Uncultured *Legionella* spp. in Treated Wastewater Effluent and Its Impact on Human Health (SCA.Re.S Project)

**DOI:** 10.3390/pathogens13090786

**Published:** 2024-09-12

**Authors:** Osvalda De Giglio, Giusy Diella, Francesco Bagordo, Antonella Francesca Savino, Angelantonio Calabrese, Mariavirginia Campanale, Francesco Triggiano, Francesca Apollonio, Valentina Spagnuolo, Marco Lopuzzo, Tiziana Grassi, Maria Clementina Caputo, Silvia Brigida, Federica Valeriani, Vincenzo Romano Spica, Maria Teresa Montagna

**Affiliations:** 1 Interdisciplinary Department of Medicine, University of Bari Aldo Moro, Piazza G. Cesare 11, 70124 Bari, Italy; giusy.diella@uniba.it (G.D.); francesco.triggiano@uniba.it (F.T.); francesca.apollonio@uniba.it (F.A.); valentina.spagnuolo@uniba.it (V.S.); marco.lopuzzo@uniba.it (M.L.); mariateresa.montagna@uniba.it (M.T.M.); 2 Department of Pharmacy–Pharmaceutical Sciences, University of Bari Aldo Moro, Via Orabona 4, 70125 Bari, Italy; francesco.bagordo@uniba.it; 3 Hygiene Section, Azienda Ospedaliero Universitaria Policlinico di Bari, Piazza Giulio Cesare 11, 70124 Bari, Italy; antonellasavino8@yahoo.it; 4 National Research Council of Italy (CNR), Water Research Institute (IRSA), Via F. De Blasio, 5, 70132 Bari, Italy; angelantonio.calabrese@ba.irsa.cnr.it (A.C.); maria.caputo@ba.irsa.cnr.it (M.C.C.); 5 Engeo soc. cop. a.r.l., 70122 Bari, Italy; virginia.campanale@gmail.com; 6 Department of Precision and Regenerative Medicine and Ionian Area (DiMePre-J), University of Bari Aldo Moro, 70121 Bari, Italy; 7 Department of Experimental Medicine, University of Salento, Via Monteroni 165, 73100 Lecce, Italy; tiziana.grassi@unisalento.it (T.G.); silvia.brigida@unisalento.it (S.B.); 8 Department of Movement, Human, and Health Sciences, University of Rome “Foro Italico”, 00135 Rome, Italy; federica.valeriani@uniroma4.it (F.V.); vincenzo.romanospica@uniroma4.it (V.R.S.)

**Keywords:** wastewater treatment plant, *Legionella* non-*pneumophila*, detection methods, viability, groundwater

## Abstract

Wastewater treatment plants (WWTPs) provide optimal conditions for the environmental spread of *Legionella*. As part of the Evaluation of Sanitary Risk Related to the Discharge of Wastewater to the Ground (SCA.Re.S) project, this study was conducted to evaluate the presence of *Legionella* in WWTP effluent and in groundwater samples collected from two wells located downstream from the plant. The samples were analyzed to determine the concentrations of *Legionella* spp using the standard culture-based method and molecular techniques, followed by genomic sequencing analysis. *Legionella* was detected only with the molecular methods (except in one sample of effluent positive for *L. pneumophila* serogroup 6), which showed viable *Legionella pneumophila* and *L.* non-*pneumophila* through the use of free DNA removal solution in both the effluent and groundwater, with concentrations that progressively decreased downstream from the plant. Viable *L. pneumophila* appeared to be slightly more concentrated in warm months. However, no significant differences (*p* ≥ 0.05) in concentrations between cold and warm months were observed. A genotypic analysis characterized the species present in the samples and found that uncultured *Legionella* spp, as yet undefined, constituted the prevalent species in all the samples (range 77.15–83.17%). WWTPs play an important role in the hygienic and sanitary quality of groundwater for different uses. The application of *Legionella* control systems during the purification of effluents is warranted to prevent possible outbreaks of legionellosis.

## 1. Introduction

*Legionella* is a Gram-negative bacterium that colonizes natural (e.g., rivers, lakes, and ponds) and man-made water environments (e.g., potable water systems, cooling towers, and fountains), including the water systems of community and hospital facilities [1,2,3,4]. The genus *Legionella* currently includes over 65 species and more than 70 serogroups [5] that replicate between 25 and 45 °C. They can survive at temperatures ranging from 5.7 to 63 °C, especially if the water is stagnant, with an optimal growth temperature of 35 ± 2 °C [6,7]. The role of protozoa (e.g., *Acanthamoeba*, *Naegleria*, and *Hartmannella*) as reservoirs for the multiplication of *Legionella* is crucial for its survival and resistance to high temperatures and biocides [3,8].

Following the inhalation of aerosols containing *Legionella*, humans can display various clinical forms of legionellosis, from severe pneumonia known as Legionnaires’ disease to the flu-like illness known as Pontiac fever.

In Italy, the incidence of legionellosis was 51.9 cases per million inhabitants in 2022, 82.5% of which were community-acquired, 2.9% nosocomial, and 14.6% due to other types of exposure. This constituted an increase relative to the previous year (46.0/1,000,000) and a return to pre-COVID-19 pandemic levels. The National Reference Laboratory (Rome, Italy) collected a total of 21 clinical strains of *L. pneumophila* isolated from patients by culture methods. The most frequent serogroup was *L. pneumophila* sg 1, while three isolates belonged to *L. pneumophila* sg 2, sg 5 and sg 8, respectively. In the same year, in the Apulia region of southern Italy, the incidence of legionellosis was 27.1 cases per million inhabitants. Particularly, of the reported 106 cases of legionellosis, 84.9% were community-acquired [9].

According to the World Health Organization, of all waterborne pathogens, *Legionella* is responsible for the greatest health burden in the European Union [10]. Therefore, the new European Drinking Water Directive 2020/2184 [10], transposed in Italy on Legislative Decree 18/23 [11], includes *Legionella* among the microbiological parameters to be detected in the potable water of healthcare and community facilities.

In the last decade, culture-independent techniques, such as PCR and DNA sequence analyses, have shown the presence of a large variety of *Legionella* types [12,13].

Although over 80% of human cases are caused by *L. pneumophila* sg 1, the remaining poorly documented cases have been attributed to *L. pneumophila* non-sg 1 and *Legionella* non-*pneumophila* (e.g., *L. micdadei*, *L. bozemanii, L. longbeachae, L. dumofii, L. feeleii,* and *L. anisa*), some of which were previously considered typically environmental species because they are widespread in water reservoirs [14,15].

Although *Legionella* contamination is frequently reported in drinking water systems [2,3,7,16], there have been few studies of *Legionella* contamination in natural environments [1,17], especially in groundwater used for drinking or irrigation purposes [18]. Moreover, few authors have considered the higher biological risk to humans in workplaces in which *Legionella*-contaminated water is used for purposes other than drinking, such as irrigation systems, and wastewater treatment plants (WWTPs) [19,20].

Previous research by us and other authors [21,22] has reported that WWTPs sometimes fail to remove all chemical and microbiological contaminants, making these water resources detrimental to human health. For this reason, Italian Legislative Decree 152/06 [23] requires the analysis of the chemical and microbiological entities in the effluent released from WWTPs, which once discharged onto soil, reaches the groundwater through the vadose zone. In Italy, this decree [23] regulates the levels of chemical parameters and specifies only *Escherichia coli* among the microbiological agents allowed in urban and industrial wastewater discharged onto soil.

These microbiological entities do not include *Legionella*, although during the wastewater treatment process, aerosols are formed, which may disperse *Legionella* from WWTPs into the environment [20]. In these plants, the combination of an ideal temperature, the availability of oxygen and organic nitrogen, and the presence of protozoa provides an optimal environment for the proliferation of *Legionella* [8], which has been detected with both culture- and molecular-based methods [20,24,25,26]. Because several authors [27,28] have reported cases of Legionnaires’ disease associated with WWTPs, it is necessary to assess the risk of *Legionella* emissions from WWTPs [20].

In the Apulia region, the Apulian Aqueduct [29] provides the regional water supply and manages 184 WWTPs, more than half of which are located in the Southern Salento area. These WWTPs release effluents directly onto the soil, which then infiltrate the underground karst and fissured or porous layers constituting the vadose zone. Therefore, in 2019, the Apulia Regional Government initiated the “Evaluation of Sanitary Risk Related to the Discharge of Wastewater to the Ground (SCA.Re.S)” project to investigate the chemical and microbial contaminants from treated wastewater discharged into porous rock formations [30] and their implications for groundwater quality. Part of the results of this project, concerning the chemical and fecal contamination of groundwater, have already been reported in previous studies [21,30].

The aims of this study were i) to investigate the contamination of groundwater by *Legionella* resulting from the discharge of treated wastewater into porous rock; ii) to compare the results obtained for *Legionella* spp. when detected with culture- and molecular-based methods; and iii) to conduct a phenotypic and genotypic analysis of uncultured *Legionella* spp. in WWTP effluent and groundwater.

## 2. Materials and Methods

### 2.1. Study Design

**Figure 1:**
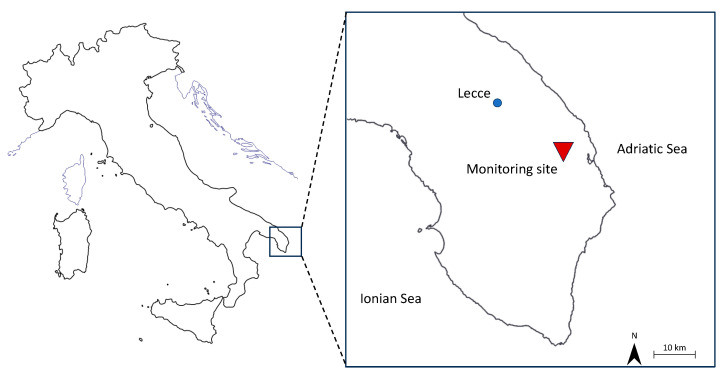
The study area, including the wastewater treatment plant and monitoring wells, on the Salento peninsula, Apulia, Italy.

**Figure 2:**
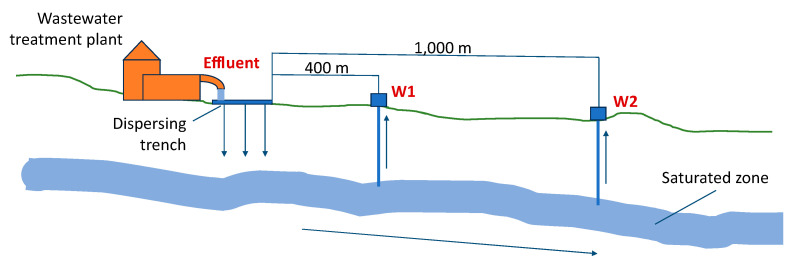
Schematic representation of the wastewater discharge path from the wastewater treatment plant into draining trenches and then to downgradient monitoring wells W1 (400 m) and W2 (1000 m).

The monitoring site was in the central–eastern area of the Salento peninsula, in the southern part of the Apulia region, Italy (Figure 1). It included a WWTP that receives urban wastewater from two small municipalities in the province of Lecce, with a total population of approximately 12,000 inhabitants. This plant processes wastewater with primary treatments, including screening and sand separation; secondary treatments, such as oxidation, denitrification, and secondary sedimentation; and finally, disinfection by chlorination. The effluent is discharged into dispersing trenches dug in the porous rock formations that constitute the unsaturated zone of the aquifer [30]. The present study involved the detection of *Legionella* spp. in the WWTP effluent and in groundwater sampled from two monitoring wells located 400 m (W1, well 1) and 1000 m (W2, well 2) downstream from the plant (Figure 2). There were no anthropogenic activities (e.g., irrigation sprinklers) in the area between the dispersing trenches and the monitoring wells that could influence the groundwater quality.

The samples were analyzed by culture- and molecular-based methods by qualitative and quantitative real-time PCR (Section 2.3). Samples (with DNA concentrations > 0.300 ng/µL) that tested positive by real-time PCR and negative by culture-based methods for *Legionella* spp were subjected to genomic sequencing analysis of 16 S rDNA gene (Section 2.4) to identify the *Legionella* species.

### 2.2. Water Sampling

Water sampling was conducted seasonally from May 2022 to April 2023, for a total of 12 sampling sessions, between 09:00 and 12:00, in calm weather with no rain. At each site (effluent, W1, and W2), 3 L of water was collected in sterile containers according to the procedures used in a previous study [18,30], for a total of 12 samples. At the time of sampling from the effluent, the temperature and concentration of free chlorine were detected in situ. The latter was measured by the N,N-diethyl-1,4-phenylenediamine sulphate (DPD) method using the Hanna Instruments HI97710 photometer (HANNA Instruments, Kehl am Rhein, Germany) with the corresponding assay kit (HANNA Instruments, Kehl am Rhein, Germany). The samples were tested for *Legionella* with culture-based methods (1 L) according to ISO 11731:2017 [31] to detect colony-forming units (CFU) and molecular PCR technique (2 L) according to AFNOR (Association Française de Normalisation) BRD (Biorad) 07/15-12/07 [32] and AFNOR BRD 07/16-12/07 [33] to detect traces of viable and non-viable DNA (see Section 2.3), respectively. The samples were transported to the laboratory at room temperature and protected from light.

### 2.3. Culture-Based and Molecular Analyses by Real-Time PCR

The culture-based analysis was conducted according to ISO 11731:2017 [31]. The water samples (1 L) were filtered through 0.2 µm isopore polycarbonate membranes with a 45 mm diameter (Millipore Corporation, Bedford, MA, USA), suspended in 10 mL of the same water sample, scraped, and vortexed. Each sample was analyzed both without any treatment and after heat or acid treatment. Specifically, an aliquot (5 mL) of the suspension was incubated at 50 ± 1 °C for 30 ± 2 min in a water bath for the heat treatment, and 1 mL was incubated with 9 mL of buffered HCl-KCl solution (pH 2.2) for 5.0 ± 0.5 min for the acid treatment. After each procedure, 0.1 mL of culture was seeded on plates containing *Legionella* selective agar (GVPC, Biolife, Milan, Italy) containing glycine, vancomycin, polymyxin, and cycloheximide (antibiotics that suppress the growth of the background flora present in water samples) and simultaneously on *Legionella* BCYE agar base (Biolife) containing buffered charcoal and yeast extract and incubated at 36 ± 1 °C for 10 days. Suspected positive colonies were subcultured on BCYE (containing l-cysteine) and BCYE without l-cysteine (Biolife). Colonies that only grew on BCYE agar plates with l-cysteine were considered *Legionella* spp. They were confirmed with serological identification, before with a latex agglutination test by polyvalent (Biolife) and then using monovalent antisera (Biogenetics Srl, Tokyo, Japan). Samples with bacterial concentrations ≥ 100 CFU/L were considered positive [31].

The molecular analysis by real-time PCR was conducted separately for *Legionella* spp. and only for *L. pneumophila* according to AFNOR BRD 07/15-12/07 [32] and AFNOR BRD 07/16-12/07 [33] protocols, respectively, and validated according to ISO 12189:2019 [34] and NF T90-471 [35]. DNA was extracted from each water sample (1 L) with the Aquadien DNA Extraction and Purification Kit (Bio-Rad, Hercules, CA, USA) according to the manufacturer’s protocol. Particularly, 1 L of water was filtered through a 0.45 µm polycarbonate filter of 45 mm diameter (Millipore), and processed for total DNA (viable/non viable cells) with qualitative and quantitative real-time PCR. The other 1 L was filtered through another 0.45 µm polycarbonate filter of 45 mm diameter and then incubated with 40 µL of iQ-Check^®^ free DNA removal solution for *Legionella* (FDRS) (Bio-Rad) for 30 min at 37 °C before bacterial cell lysis to ensure the evaluation of only intact and viable cells. The reagent Aquadien Extraction kit R1 (500 µL) was added to the membrane filters, followed by the lysis solution from the Aquadien Kit (Bio-Rad). The DNA was extracted according to the manufacturer’s protocol. The extracted DNA (5 µL) from each sample was diluted 10^0^ and 10^−1^ and subjected to real-time PCR amplification with two different iQ-Check^®^ Screen *Legionella* real-time PCR detection kits (Bio-Rad) validated AFNOR (one for *Legionella* spp. [32] and the other only for *L. pneumophila*, respectively) [34] on both the FDRS-treated and untreated samples. Quantitative PCR was then performed on the positive samples with the iQCheck^®^ Quanti real-time PCR quantification kits separately for *Legionella* spp. and only for *L. pneumophila*, respectively. These kits amplified a structural gene (rRNA5S) for *Legionella* spp and a part of the virulence gene sequence (mip) that can only be recognized for *L. pneumophila*. The genomic units per litre (GU/L) were determined with a CFX96 Touch real-time PCR detection system (Bio-Rad) and CFX Manager™ Software version 4.1.2433.1219 (Bio-Rad). For qualitative detection of *Legionella* spp, samples were considered positive when Cq ≥ 10 (Cq, quantification cycle) and the internal control was not amplified. For quantitative detection of *Legionella* spp, the samples were considered positive when Cq ≥ 10 and internal control Cq ≤ mean Cq Qs + 3σ, where the mean Cq Qs (quantitative standard) is the mean of values of all Cq of Qs internal control (HEX), and σ is the standard deviation.

### 2.4. Genomic Sequencing Analysis

All the concentrations of DNA extracts were determined with a Qubit 4™ Fluorometer (Thermo Fisher Scientific, Wilmington, DE, USA) with the Qubit™ 1 × dsDNA HS Assay Kit (Thermo Fisher Scientific). Samples with DNA concentrations > 0.300 ng/µL were subjected to amplicon sequencing of 16S rRNA gene fragments. The 16S rRNA gene sequences of the sample strains were compared with those of *Legionella* reference strains in the National Center for Biotechnology Information (NCBI) GenBank database version 2.16.0 to identify specific strains (>97% sequence identity) [36]. A bioinformatic neighbor-joining analysis was used to generate phylogenetic trees from which to infer the relationships between the reservoir isolates detected in this study and the reference strains.

#### 2.4.1. Illumina MiSeq Library Design, Preparation and Bioinformatic Analysis

The preparation and construction of next-generation sequencing (NGS) libraries were based on a dual-indexing strategy adapted from previously described protocols [37,38,39]. A two-step PCR approach was used to prepare the libraries. The first PCR with high-purity salt-free custom-synthesized primers (purified by Eurofins MWG Operon, Ebersberg, Germany), which complemented the 16S rRNA gene regions, introduced sample barcodes and complementary adapter regions. The variable V3 and V4 regions of the 16S rRNA gene (bp 789–984) were amplified with the universal primers. All reactions were performed in 50 µL reaction mixtures. The target-specific PCR mix comprised 0.1 mM each dNTP (Bioline, Luckenwalde, Germany), 0.4 mM MgCl_2_, 1 × PCR reaction buffer, 0.03 U of HotStar Taq Polymerase (Qiagen), 0.4 µM each primer, and 2 ng of DNA. Amplification was performed in the 96-well iCycler iQ™ Real-Time PCR Detection System (Bio-Rad). For multiplexing PCR, 2 µL of the target-specific PCR product was used as the template. The reaction mixture also contained 0.1 mM each dNTP (Bioline), 0.75 mM MgCl_2_, 1 × PCR reaction buffer, 0.03 U of HotStarTaq DNA polymerase (Qiagen), and 0.4 µM each primer.

The second PCR, a non-target-specific assay, integrated the complementary sequences to the flow-cell-binding sites, and the addition of an index to the reverse primer allowed for a multiplex analysis of the samples. The 16S rRNA gene amplicons obtained were selected for size with electrophoresis on 2% agarose gel pre-stained with GelRed^®^ Nucleic Acid Gel Stain (Biotium, Hayward, CA, USA). Bands of the expected target length were extracted under a UV light box and then recovered with the QIAquick gel extraction kit (Qiagen, Hilden, Germany), for which the manufacturer’s specifications were slightly modified. Buffer QG (1.5 mL) was added to the gel band and incubated at room temperature for 15 min [40]. Next, 250 µL of isopropanol (AppliChem, Darmstadt, Germany) was added, and the mixture was vortexed, placed in a QIAquick spin column, and centrifuged at 13,000 rpm for 1 min. The remaining steps to obtain clean library amplicons in 30 µL of EB buffer were performed according to the manufacturer’s instructions. The DNA concentrations of the extracted amplicons were determined with a Victor X3 2030 Multimode Multilabel Plate Reader (Perkin Elmer, Germany) and the Quant-iT™ PicoGreen™ dsDNA Assay Kit (Life Technologies, Hillsboro, OR, USA) to obtain equimolar amounts of DNA with unique indices. The pooled samples were then purified with the MinElute PCR Purification Kit (Qiagen), according to the manufacturer’s instructions. The molarity of the libraries was determined and the library fragment size was confirmed with an Agilent 2100 Bioanalyzer System. The resulting libraries were sequenced in the Genome Analysis Department of the Helmholtz Centre for Infection Research (Braunschweig, Germany) using the Illumina MiSeq platform version 1.0.1, generating paired-end 250-bp reads.

Raw sequence data were processed using an in-house pipeline and various software tools to evaluate the quality of the raw sequence data (FASTA/Q Information tools, Mothur) [41]. All data sets were rigorously screened to remove low-quality reads (short reads > 200 nucleotides, zero-ambiguity sequences). Demultiplexing was performed to remove PhiX sequences and sort sequences; moreover, to minimize sequencing errors and ensure sequence quality, the reads were trimmed based on the sequence quality score using Btrim (with an average quality score of 30 from the ends; reads than 200 bp were removed after end trimming) [42]. OTUs (operational taxonomic units) were clustered at a 97% similarity level, final OTUs were generated based on the clustering results, and taxonomic annotations of individual OTUs were based on representative sequences using RDP’s 16S Classifier 2.5. Observed OTUs were defined as observed species. A level of 97% sequence identity is often chosen as representative of a species (versus 95% for a genus) [43]. The sequence reads were also analyzed in the cloud environment BaseSpace through the 16S Metagenomics app (version 1.0.1; Illumina); the taxonomic database used was the Illumina-curated version [43].

#### 2.4.2. DNA Analyses

To better detect and analyze the presence of *Legionella* spp. during the study period, we performed a BLASTN search to evaluate the similarity of the 16S rRNA gene sequences deposited in the GenBank database. All the sequences were included in the ARB software package version 7.0 and the SILVA database version 138.2 and were optimally aligned with the fast-aligner tool. The number of operational taxonomic units (OTUs) and the total species richness were determined with the DOTUR (distance-based OTU and richness) software program [44]. OTUs were defined as sequences with at least 97% similarity. Phylogenetic analysis was performed using the fast-aligner tool. A phylogenetic tree of the 16S rRNA gene sequences was constructed by the neighbor-joining method.

### 2.5. Statistical Analysis

The results of the molecular investigations were entered into a database and statistically processed with the MedCalc software version 12.3 (MedCalc Software bvba, Ostend, Belgium). For each group of data, the arithmetic mean, standard error (SE), and maximum and minimum values were calculated.

A Pearson correlation analysis was conducted to verify any relationship between the concentrations of viable *Legionella* and the temperatures or concentrations of free chlorine detected in the effluent.

The analytical results were categorized into two groups based on the mean monthly temperatures recorded in the province of Lecce and reported by the Italian Air Force Meteorological Service [45]. The first group contained the results obtained from sampling during the cold months, November–April (average minimum temperature 5.9± 0.6 °C; average maximum temperature 15.4 ± 0.7 °C). The second group contained the samples collected during the warm months of May–September (average minimum temperature 15.7 ± 0.9 °C; average maximum temperature 27.7 ± 1.2 °C).

To compare the concentrations of *Legionella* spp. and *L. pneumophila* at the different sampling points and in the two periods of the year, the Shapiro–Wilk test was initially used to verify the normal distribution of the values in each group. If the distribution was normal, the differences were assessed with a one-way ANOVA. However, if the distribution was non-normal, the Mann–Whitney test was used. A *p*-value < 0.05 was considered to indicate a statistically significant difference.

## 3. Results

### 3.1. Culture-Based And Molecular Analyses by Real-Time-PCR

The culture-based method gave negative results for *Legionella* spp. in all but one sample. *L. pneumophila* sg 6 was detected in only one sample, at a concentration of 300 CFU/L, specifically in the July effluent.

**Figure 3:**
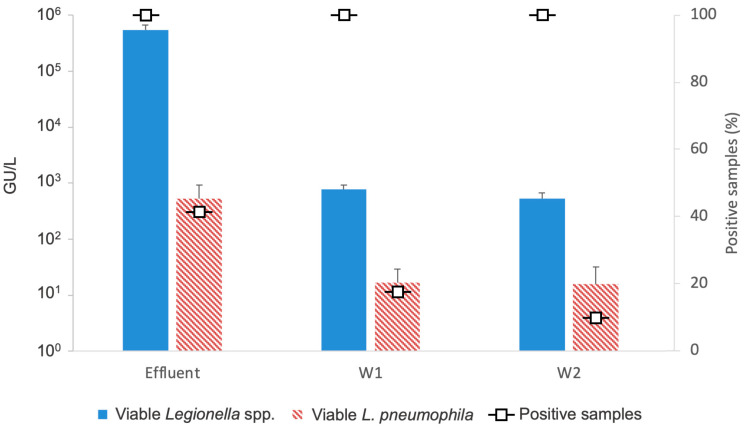
Rate (%) of positive samples and viable cell concentrations of *Legionella* spp. and *L. pneumophila*, collected from the effluent of WWTP, W1 (well 1), and W2 (well 2).

The molecular method detected viable cells of *Legionella* spp. in 100% of the samples, with mean concentrations of 5.44 × 10^5^ ± 1.27 × 10^5^ GU/L (min = 1.70 × 10^3^ GU/L; max= 1.51 × 10^6^ GU/L) in the WWTP effluent, 7.72 ×10^2^ ± 1.64 × 10^2^ GU/L (min = 9.30 × 10^1^ GU/L; max= 9.88 × 10^3^ GU/L) in the W1 sample, and 5.36 × 10^2^ ± 1.39 × 10^2^ GU/L in the W2 sample (min = 1.60 × 10^2^ GU/L; max= 1.43 × 10^3^ GU/L) (Figure 3).

The genome of viable and non-viable cells of *L. pneumophila* was found in 91.7% of the samples from effluent, in 36.1% of the samples from W1, and in 10% of the samples from W2 (in the remaining percentage of samples positive for *Legionella* spp., the presence of *L.* non-*pneumophila* was detected). However, viable *L. pneumophila* was detected in 41.7% of samples from effluent, in 18.2% of the samples from W1, and in 10.0% of the samples from W2, with a mean concentration of 5.27 × 10^2^ ± 3.97 × 10^2^ GU/L (min = 0 GU/L; max = 4.80 × 10^3^ GU/L), 1.68 × 10^1^ ± 1.24 × 10^1^ GU/L, and 1.60 × 10^1^ ± 1.60 × 10^1^ GU/L (min = 0 GU/L; max= 1.60 × 10^2^ GU/L), respectively (Figure 3).

The mean temperature recorded in the WWTP effluent at the time of sampling was 18.8 ± 0.8 °C. The correlation between the concentration of *Legionella* spp. or *L. pneumophila* and effluent temperature was found to be statistically non-significant (*p* > 0.05), with a weak negative correlation (R = −0.49) for *Legionella* spp. and a weak positive correlation (R = 0.14) for *L. pneumophila*. The mean concentration of free chlorine in the effluent was 0.06 ± 0.01 ppm. As with the previous analysis, the relationship between free chlorine concentration and the concentration of *Legionella* spp. as well as *L. pneumophila* was found to be very weak (R = 0.23 and R = −0.49, respectively) and not statistically significant (*p* > 0.05).

**Figure 4:**
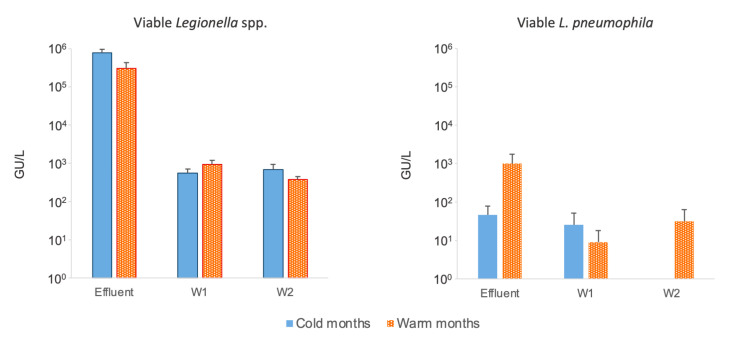
Average concentrations ± SE of viable *Legionella* spp. and *L. pneumophila* in WWTP effluent W1 (well 1) and W2 (well 2) recorded in cold months (November 2022–April 2023) and warm months (May–October 2022).

The presence of viable *Legionella* spp. was identified in 88.9% of the samples collected during the cold months (100% in effluent, 83.3% in W1, and 83.3% in W2) and in 94.4% of the samples collected during the warm months (100% in effluent, 100% in W1, and 83.3% in W2) (Figure 4). However, no statistically significant differences (*p* ≥ 0.05) were observed in the concentrations of *Legionella* spp. between the cold and warm months. The presence of viable *L. pneumophila* was identified in 16.7% of the samples collected during the cold months (33.3% in effluent, 16.7% in W1, 0.0% in W2) and in 27.8% of the samples collected during the warm months (50.0% in effluent, 16.7% in W1, 16.7% in W2). The molecular analysis revealed a notable though not statistically significant (*p* > 0.05) increase in the concentration of *L. pneumophila* during the warm months in the effluent and in W2.

### 3.2. NGS Results and Identification of Legionella spp.

After quantification of the DNA concentrations in the genic extracts, six samples (one/month from March to October, except August and September) of effluents from the WWTP and one sample from a monitoring well (W1) located downstream from the treatment plant were selected for sequencing analysis.

In the samples analyzed, several OTUs correlated to the bacterial community with a constant trend in the months analyzed, with a minimum value in July (426,712 OTUs) and a maximum value in April (590,974 OTUs). In June, the sample from W1 contained 447,018 OTUs in the bacterial community, which was fewer than in the effluent samples from the treatment plant, which contained a value of 579,845 OTUs.

Genotypic analysis detected 41.75–47.17% *Legionella* spp. DNA in the WWTP effluent samples and 41.82% in the sample taken from W1.

**Figure 5:**
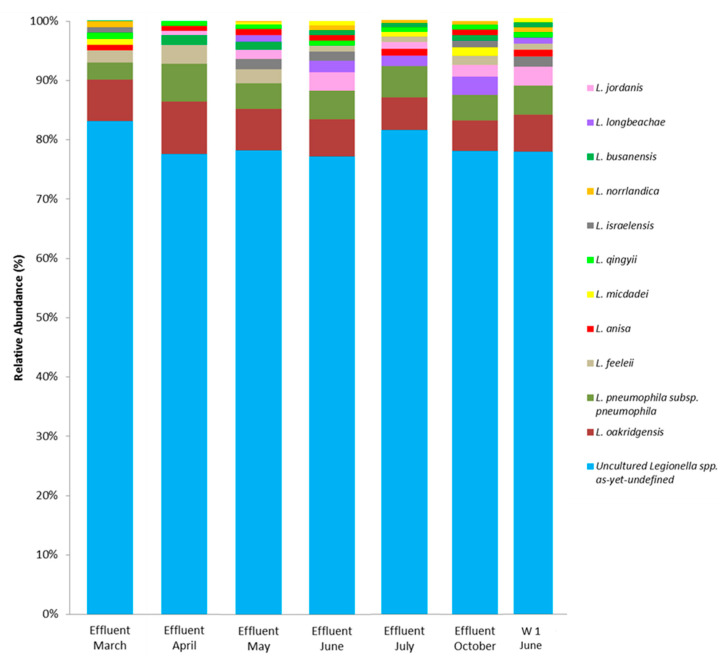
*Legionella* spp.’s composition in water samples assessed by sequencing 16S rRNA gene V3–V4 domain amplicon with Illumina MiSeq (similarity > 97%).

Characterization of the *Legionella* species showed that uncultured as-yet-undefined *Legionella* spp constituted the highest proportion in the effluent (range 77.15–83.17%) and in the W1 sample (77.55%) (Figure 5). Specifically, the Table 1 shows the average (%) occurrence of uncultured as-yet-undefined *Legionella* clones.

With regard to uncultured identified *Legionella* species, the proportions of *L. oakridgensis* (5.12–8.80%), *L. pneumophila* subsp. *pneumophila* (2.97–6.40%), *L. feeleii* (0.98–3.20%), *L. jordanis* (0.80–3.12%), *L. longbeachae* (1.06–3.00%), *L. busanensis* (0.70–1.60%), *L. israelensis* (0.62–1.75%), *L. micdadei* 0.47–1.40%), *L. anisa* (0.80–1.12%), *L. qingyii* (0.75–0.99%), and *L. norrlandica* (0.18–0.98%) were determined in effluents (Figure 5).

Similarly, the proportions of *L. oakridgensis* (6.30%), *L. pneumophila* subsp. *pneumophila* (4.84%), *L. jordanis* (3.21%), *L. israelensis* (1.69%), *L. anisa* (1.10%), *L. feeleii* (1.04%), *L. longbeachae* (1.00%), *L. qingyii* (0.92%), *L. busanensis* (0.80%), *L. norrlandica* (0.80%), and *L. micdadei* (0.75%) were also determined in the W1 sample (Figure 5).

**Figure 6:**
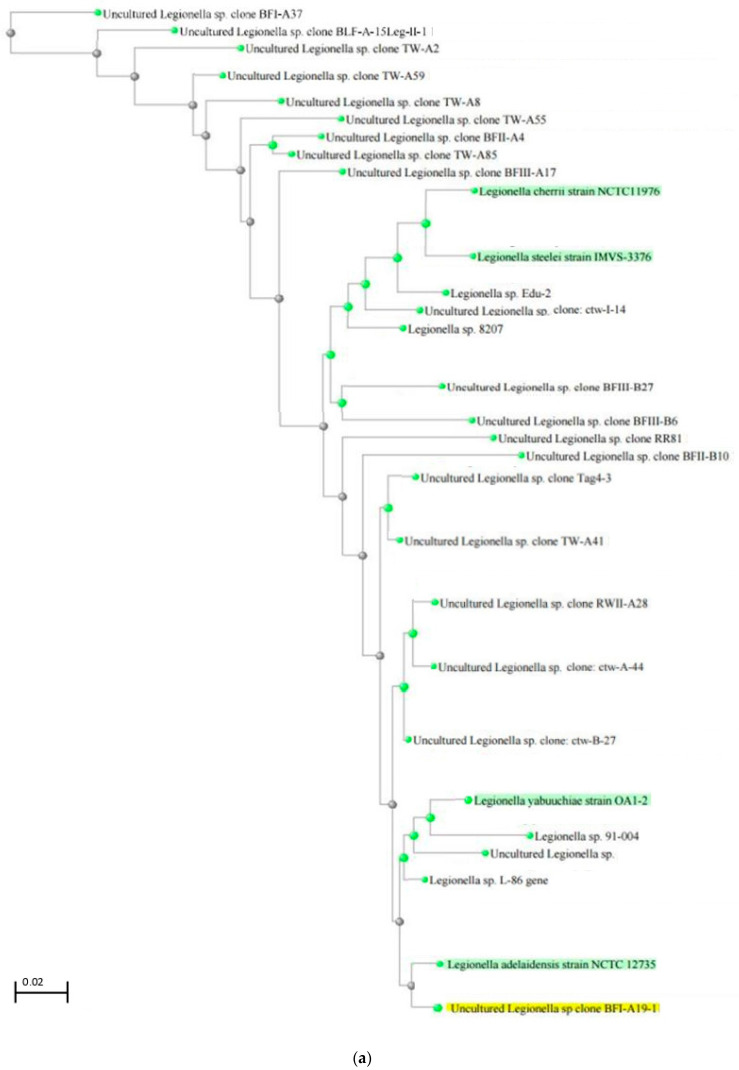

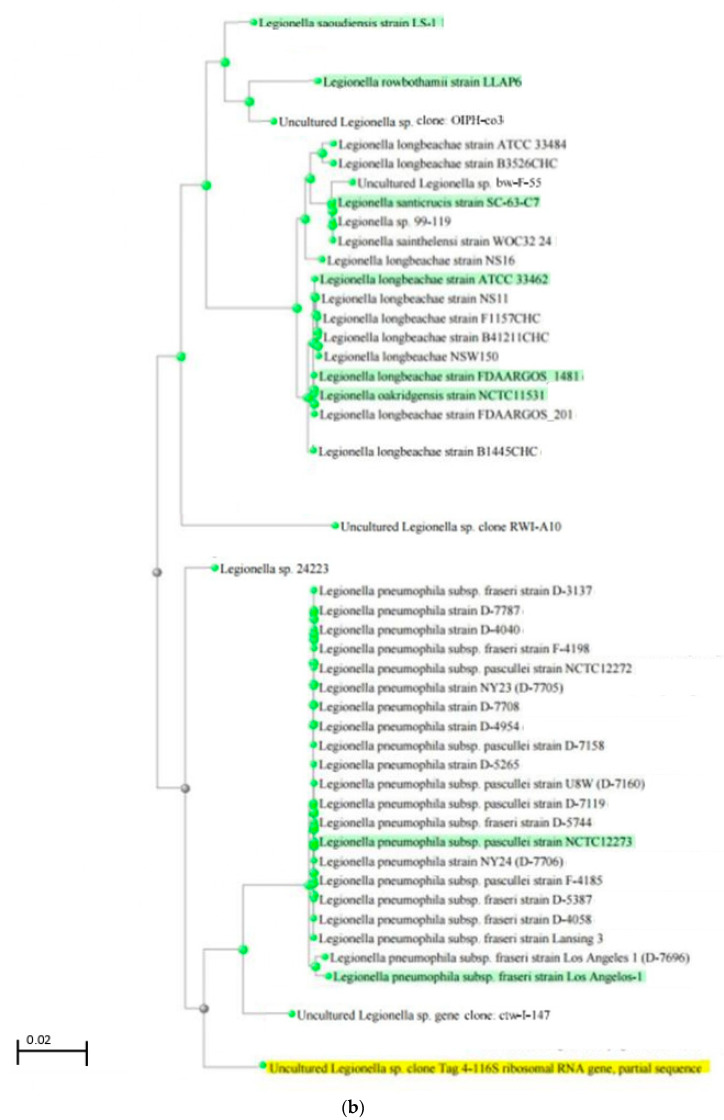
(**a**) NCBI BLASTN phylogenetic tree showing the similarities (>97%) between the uncultured Legionella sp. clone BFI-A19-1 (in yellow) and known Legionella species (in green), determined with 16S rDNA sequencing. The scale bar corresponds to 0.02 substitutions per nucleotide position. (**b**) NCBI BLASTN phylogenetic tree showing the similarities (>97%) between uncultured Legionella sp. clone Tag 4-1 (in yellow) and known *Legionella* species (in green), determined with 16S rRNA sequencing. The scale bar corresponds to 0.02 substitutions per nucleotide position.

Based on these results, a basic phylogenetic analysis was performed to better understand the possible proximity of the identified uncultured species to known *Legionella* species. The data obtained from the NCBI database indicated that among the six most abundant species (Table 1), four were phylogenetically distant from known *Legionella* species. However, the uncultured *Legionella* spp. clone BFI-A19-1 was phylogenetically close to *L. adelaidensis* (Figure 6a), and uncultured *Legionella* sp. clone Tag 4-1 was phylogenetically close to *L. pneumophila* (Figure 6b). These data should be validated with further in-depth phylogenetic analyses.

## 4. Discussion

It is widely recognized that the current limited availability of water requires that the quality of natural water resources—such as lakes, rivers, and groundwater–be preserved for both drinking and irrigation. To date, several studies globally [8,12,24,27,28,46,47] but few conducted in Italy [48,49] have demonstrated the presence of *Legionella* in wastewater and groundwater, although most have used non-culture-dependent methods, such as qPCR. To the best of our knowledge, this is the first year-long study in Italy of the abundance and genotypic diversity of *Legionella* in wastewater and groundwater using molecular methods, ranging from qPCR to genomic sequence analysis of the 16 S rDNA gene (bp 789–984).

With regard to molecular methods, our results showed a progressive reduction in the presence of *L. pneumophila* viable and non-viable cells and concentration of *Legionella* spp. and *L. pneumophila* viable cells (Figure 3) in water samples collected as the distance from the WWTP increased. Our previous study [30] showed that porous rocks are more effective than others (e.g., karst or fractured rocks) in retaining microorganisms from treated wastewater. Porous rock acts as a filter, promoting the removal of contaminants through chemical (e.g., grain-surface coatings, ionic strength of the pore water), physical (e.g., grain and pore sizes), and biological mechanisms (e.g., cell viability, cell size and shape, biofilm formation) [1,50,51,52]. However, because the survival rate of *Legionella* in soil is high, its slow transport does not completely reduce the risk of groundwater contamination [53].

The positivity for viable *Legionella* spp. and viable *L. pneumophila* was observed to be higher in the warm months than in the cold months, suggesting a possible correlation between temperature and the viability of these bacteria. However, no significant differences were observed in the pattern of *Legionella* concentrations between the two time periods (*p* ≥ 0.05). This could indicate that the depth of the wells (natural environment) guaranteed a certain stability of water temperature and the constant proliferation and spread of the bacterium, unlike artificial water systems. It should be mentioned that although the molecular analysis was performed according to AFNOR BRD 07/15-12/07 and AFNOR BRD 07/16-12/07 standards, the use of 0.45 µm membrane filters compared to the 0.2 µm membrane filters used in the culture-based method could underestimate the presence of *Legionella* [54]. Therefore, future research should take into account this limitation of the study.

To date, few studies have detected the presence of *Legionella* in wastewater or groundwater with a culture-based method [18,20,26,46,55]. In the present study, only one sample of WWTP effluent was positive for *L. pneumophila* sg 6 when a culture-based method was used, whereas the molecular methods detected viable *L. pneumophila* and *L.* non-*pneumophila* in all the samples analyzed. These results are in agreement with a previous study we conducted in the same area [18] that revealed the presence of *Legionella* spp. by qPCR in groundwater that tested negative using the culture-based method. Although the culture-based method is the gold standard for the detection of *Legionella*, it has several limitations. First, on plates, despite pre-treatment according to ISO 11731:2017, high concentrations of other contaminant microorganisms could interfere with *Legionella* growth, making its isolation difficult. It has also been shown that the culture-based method according to ISO 11731:2017 is not very sensitive in detecting *Legionella pneumophila* and *Legionella* non-*pneumophila* at low concentrations [56], as also demonstrated in our study. In our study, moreover, according to AFNOR BRD 07/15-12/07 and AFNOR BRD 07/16-12/07 standards, the plates were incubated for only 10 days, but several papers in Italy suggest leaving the plates for at least 15 days because *L. non-pneumophila species* need more time to grow [57,58,59]. Therefore, in light of our results and in agreement with other authors [12,47], PCR-based methods and genomic sequencing analysis, when applied to effluent and W1 and W2 samples, allow the quantitative detection of *L. pneumophila*, *L.* non-*pneumophila*, and as-yet-undefined *Legionella* sequences, even at low concentrations. Moreover, the use of FDRS (non-toxic reagent) allowed us to differentiate between dead and viable cells, as well as treatments with molecules such as ethidium monoazide and propidium monoazide but with a procedure that is simpler (i.e., does not require light activation), faster, and safer for the operator’s health [18,55].

As a result, non-culture-dependent methods resolved the problem that occurs with standard culture-based methods, by detecting *Legionella* in the viable but non-culturable (VBNC) state [8,20,55]. Little is known about the physiological processes, underlying genetic and epigenetic regulation, and virulence of these cells.

Different stressors that induce the transition of *Legionella* to the VBNC state have been described, as follows: nutrient deficiency [60]; chemical treatments, such as chlorination [61]; and physical factors, such as the heat used in WWTPs [62]. Therefore, even acid or thermal pretreatment procedures according to ISO11731:2017 are likely to induce culturable cells to enter the VBNC state. This may explain the observed insensitivity and lack of reproducibility often noted with *Legionella* culture methods [62]. However, *Legionella* also replicates within natural hosts such as amoebae, which they exploit as sources of nutrition and through them some VBNC cells recover their cultivability [63]. Therefore, an understanding of *L. pneumophila*–protozoan interactions is important if we are to improve the management of water systems [3].

Genotypic and phylogenetic analyses with DNA sequencing allowed the detection of *Legionella* spp., including both well-known pathogenic species and species rarely responsible for pathology or not yet identified (Figure 5). A high diversity of *Legionella* spp. was observed in all samples, and consistently with other studies [64], the dominant *Legionella* clusters identified were most closely related to uncultured unidentified *Legionella* species. When we compared the effluent with the well water downstream from the treatment plant, similar diversities of *Legionella* species were detected.

A sequencing analysis revealed that *L. pneumophila* represented only a small fraction of all the *Legionella* species isolated, preceded by *L. oakridgensis*, the main population reported in previous studies [65], and followed by *L. feeleii* in the effluent samples and by *L. jordanis* in the well sample (Figure 5).

Although *L. pneumophila* is the most clinically important *Legionella* species, some *Legionella* non-*pneumophila* species detected in our water samples at low concentrations have been associated with human disease. *Legionella feeleii* can be considered one of the main pathogenic microorganisms among *Legionella* species, implicated in both Pontiac fever and Legionnaires’ disease [66]; *L. oakridgensis* (the only *L. species* capable of growing on medium without cysteine) is less virulent than *L. pneumophila* but can cause Legionnaires’ disease, although rarely [67]; *L. jordanis* was first isolated from a bronchoalveolar lavage specimen from a patient with an indolent lower respiratory tract infection associated with constitutional symptoms in Canada [68]. Finally, *L. anisa*, *L. bozemanii*, and *L. longbeachae* are well-known human pathogens based on their isolation from clinical material [69,70]. On the contrary, *L. busanensis, L. norrlandica*, *and L. qingyii* are new environmental species that are not currently associated with clinical infections and therefore would not pose a risk to humans [5]. *Legionella israelensis* has been isolated from oxidation ponds and fishponds in Israel, but very little detailed information on its ecological characteristics is available [13].

The high percentage of undefined *Legionella* sequences (Figure 5; Table 1) detected suggests that studies using PCR-based methods and phylogenetic trees (Figure 6a,b) will lead to a large increase in the number of species within the genus *Legionella* [12]. Furthermore, isolating these different species will aid in better understanding their persistence in aquatic environments and determining their pathogenicity.

Interestingly, in our study, *Legionella* was detected in all samples with a molecular-based method, in some cases even at high genomic concentrations, inconsistent with the culture-based method. These data raise some critical issues with respect to new Italian Legislative Decree 18/23, which provides that any positive result for *Legionella* obtained with molecular methods must be confirmed with a culture-based method. Consequently, it is necessary to understand how to interpret a culture-negative sample with a high concentration of *Legionella* genome copies. The lack of correlation between the different methods highlights the need to develop a standardized method for the quantification of *Legionella* appropriate for the risk assessment and management of this human pathogen in aquatic environments, particularly artificial water systems [55].

## 5. Conclusions

Wastewater treatment plants play an important role in the decontamination of water resources. Culture-based methods are still the most widely used standard technique for the detection and quantification of viable *Legionella* in environmental samples. Molecular analyses have been shown to be sensitive investigative tools and should complement culture techniques in the detection of *Legionella*.

Because it is important to apply effective control systems to WWTP effluents to prevent possible outbreaks of legionellosis, further studies are required to clarify how to interpret and evaluate the limitations of culture-based and molecular detection methods. Moreover, the role of *L.* non-*pneumophila* species in natural and man-made aquatic environments must also be investigated with respect to the risk of legionellosis. Genotypic and phylogenetic analyses make it possible to determine both the presence of pathogenic *Legionella* species and the diversity of indigenous strains.

## Figures and Tables

**Table 1:** Average value (%) of uncultured as-yet-undefined *Legionella* species.

Uncultured as-Yet-Undefined *Legionella* Species (GenBank Accession No.)	Average Occurrence (%)
*Legionella* spp. clone AL1_5C_N1 (KM624105)	20.82
*Legionella* spp. clone Tag 4-1 (AY924177)	18.29
*Legionella* spp. clone F29/FB1 (GU979470)	17.52
*Legionella* spp. clone PmeaH2OD1 (EU249944)	14.43
*Legionella* spp. clone IL3E_N1 (KM624120)	8.59
*Legionella* spp. clone BFI-A19 (HQ111761)	7.52
Other	12.83

## Data Availability

Data are contained within the article.
